# Editorial: Exosome-based advances in endocrine and metabolic diseases: from diagnosis to therapy

**DOI:** 10.3389/fendo.2025.1664014

**Published:** 2025-08-15

**Authors:** Alok Raghav, Jamal Ahmad

**Affiliations:** ^1^ Department of Anatomy and Cell Biology, College of Medicine, Gachon University, Incheon, Republic of Korea; ^2^ University Center for Research & Development (UCRD), Chandigarh University, Chandigarh, India; ^3^ Center for Global Health Research, Saveetha Medical College and Hospital, Chennai, India; ^4^ Rajiv Gandhi Centre for Diabetes and Endocrinology, J.N Medical College, Aligarh Muslim University, Aligarh, India

**Keywords:** exosome, type 1 diabetes, metabolic disease, endocrinology, therapy, diagnosis

The dynamic interplay between exosomes and diabetes pathophysiology has increasingly attracted scientific attention, establishing exosomes not merely as passive vesicles but as active mediators of disease mechanisms, diagnostics, and therapeutics. A close examination of recent contributions to the field, as reflected in several impactful articles from *Frontiers in Endocrinology*, highlights a progressive narrative of discovery, innovation, and translational potential.

In an earlier contribution to this growing body of work, Mishra et al. explored the therapeutic potential of urinary exosomes (uE) derived from rats with diabetic kidney disease (DKD). Their study provided compelling evidence that uE carries reno-protective miRNAs, such as miR-24-3p and miR-200c-3p, which are depleted in the renal tissues of diabetic nephropathy (DN) patients but enriched in their urine. Injecting these exosomes back into diabetic rats restored renal miRNA levels and attenuated pathological changes. This was among the first demonstrations that reintroducing urinary-excreted miRNAs can therapeutically mitigate renal injury in diabetes, suggesting a novel angle in RNA-based treatment strategies for DN.

Advancing from renal complications to vascular dysfunction, Xie et al. highlighted the deleterious role of peripheral blood-derived exosomal miR-135a-3p in promoting vascular injury in Type 2 diabetes (T2D). Their study used both in silico and *in vitro* models to demonstrate that miR-135a-3p targets ATM—a key kinase involved in DNA damage repair—and alters the ErbB signaling pathway, leading to increased vascular smooth muscle cell proliferation and migration. This study provides deep insights into the mechanistic driving of miRNA in diabetes microvascular complications and poses miR-135a-3p as a potential biomarker and a therapeutic target in vascular injury.

Progressing the findings further, Gao et al. highlight the present understanding of exosomal microRNA (miRNAs) in diabetic cardiomyopathy (DCM). Their comprehensive findings summarize how secretory vesicle exosomes serve to be carriers of pathogenic signals released from stressed cardiomyocytes, contributing to impaired angiogenesis and exacerbating fibrosis in the diabetic heart. Specifically, the authors show that exosomal miRNAs, particularly those that modulate the ERK1/2 and p38MAPK mechanisms, can be leveraged to develop cardioprotective approaches. The study emphasized the potential of exosome profiling in early diabetic cardiomyopathy screening and targeted miRNA-based therapies, thus opening new windows in cardiac care for diabetic patients.

Meanwhile, a crucial observation in pediatric type 1 diabetes mellitus (T1DM) done by Bai et al. uncovers exosomal protein signatures in T1DM children. Mass spectroscopy-based proteomics was exploited by the authors to report differential protein expression in plasma-derived exosomes that play a crucial role in immune regulation, homeostasis, and cellular stress stimuli. Conspicuously, these exosomal profiles were found to be normalized in a controlled HbA1c scenario. These findings encouraged the implication of exosomal proteins as biomarkers for the early detection of disease and surrogates for therapeutic response in T1D.

At the same time, by investigating the role of beta (β) cell senescence in modulating the nature of secretory exosomes (EVs) content in T1D, Motlagh et al. supplement a newer perspective to this discussion. According to this study, senescent β-cells release EVs that are enriched in pro-inflammatory miRNAs and SASP, which aid in the advancement of the disease. This study calls for further investigations into how cellular ageing alters EV cargo and its potential in targeted clearance of senescent cells in T1D therapy by presenting EVs as modulators of immune activation.

Han et al. provide a landmark observation in which they stated that the adipose tissues derived from extracellular vesicles mediate inter-organ communication in obesity, a common prelude to type 2 diabetes in a metabolic context. The divergent role of extracellular vesicles from healthy versus obese adipose tissues was distinguished in this study. The latter banquets inflammatory and metabolic dysfunction, whereas the former encourages tissue repair and metabolic homeostasis. The authors assert that these EVs can be isolated from lipoaspirates and can be used in a therapeutic role because of their low immunogenicity and modifiability.

A study by authors Pian et al. on pancreatic intraepithelial neoplasia shows how it inaudibly strengthens the exosomal-diabetes axis. While mainly aimed at precursors of pancreatic cancer, they highlight miRNAs like miR-21 and miR-155, both of which are associated with pancreatic intraepithelial neoplasia and prior markers of diabetic inflammation. The observations here support a wider implication for miRNA-based intracellular communication for endocrine and oncological diseases, unveiling molecular currency across disease spectrums.

## Conclusion

Exosomes are observed as multifaceted contributors in the landscape of diabetes mellitus. Their protagonist role spans an eclectic spectrum from prompting renal and vascular complications to curbing immune responses, driving β-cell senescence, altering adipose tissue signaling, and contributing to tumorigenic events. With time, research has progressed from exploration of specific miRNAs to reconnoitering comprehensive proteomic and transcriptomic profiles, marking significant improvements. These intuitions offer immense promise for reforming the diagnosis and treatment of diabetes mellitus and its associated complications. There is an unrelenting necessity for standardized protocols in exosome isolation, cargo characterization, clinical-grade production at a mass level, and their application. Furthermore, a meticulous mechanistic understanding of EVs’ biogenesis in the diabetic scenario and the fabrication of targeted delivery systems for therapeutic use are crucial next phases. Despite these obstacles, the future is encouraging: as molecular gears become more polished, so does our potential to leverage these nanoscale vesicles for precise and personalized approaches to management of diabetes mellitus and its associated complications.

